# Artificial Intelligence in the Prediction of Stone-Free Status in Urinary Stone Disease Treated with Extracorporeal Shockwave Lithotripsy: A Systematic Review

**DOI:** 10.12688/f1000research.152346.2

**Published:** 2025-03-26

**Authors:** Ficky, Nur Rasyid, Widi Atmoko, Ponco Birowo

**Affiliations:** 1 Department of Urology, Faculty of Medicine, Universitas Indonesia, Jakarta, Indonesia

**Keywords:** artificial intelligence, stone-free status, urolithiasis, extracorporeal shock wave lithotripsy

## Abstract

**Background:**

Urolithiasis is one of the most common urological diseases worldwide. One of the most common therapy, extracorporeal shock wave lithotripsy (ESWL), has a high failure rate. The failure rate can be significantly reduced by identifying the candidates most likely to benefit from ESWL, for example, by using machine learning (ML) algorithms. Decision tree analysis (DTA), artificial neural networks (ANN), and random forests (RF) represent a few of the machine learning approaches employed to forecast the stone-free outcome following ESWL.

**Methods:**

219 studies were searched through six electronic databases (CENTRAL, MEDLINE, EMBASE, EBSCO, Proquest, SCOPUS). We employed the Preferred Reporting Items for Systematic Reviews and Meta-Analysis (PRISMA) guidelines and adhered to the Standards for Reporting Diagnostic Accuracy Studies (STARD). To evaluate the potential bias in all the studies, we utilized the Quality Assessment of Diagnostic Accuracy Studies (QUADAS-2) tool.

**Results:**

41,484 patients from 11 studies were included. The ML models highlight varying levels of diagnostic precision, with sensitivity spanning from 35-96%, and specificity ranging from 63-98.4%, and area under the curve falling between 0.49-0.96. It is shown in this study that the accuracy of RF and DTA in predicting stone-free status is superior than ANN.

**Conclusion:**

ML is a comparable predictive method to statistical analysis in predicting stone-free status. Random forest method and DTA are superior MLs compared to ANN. Stone size, density, and 3D texture analysis are the most important variables to be considered in the ML models and should be included in the models to ensure accuracy of stone-free status prediction.

## Introduction

Urolithiasis ranks as the third most prevalent ailment encountered in urological practice, trailing only urinary tract infections and prostate irregularities in terms of occurrence. This condition stands as one of the most widespread urological maladies worldwide, with estimated prevalence rates spanning from 1% to 13% across various regions. ^1^ The global incidence, prevalence, and composition of urinary stones exhibit notable disparities and have undergone transformations over recent decades. Specifically, prevalence rates range from 7% to 13% in North America, 5% to 9% in Europe, and 1% to 5% in Asia. ^2^ This condition’s incidence is reported to range between 5% and 10%, occurring three times more frequently in men than in women. Individuals aged 30 to 50 face a heightened risk of developing urolithiasis, and it’s noteworthy that some patients experience recurrent stone formation. Most stones are naturally expelled through urination, with the duration of this process contingent on the stone’s size and location. Spontaneous passage occurs in approximately 80% of ureteral stones smaller than 5 mm. ^3^

Various methods are employed to actively remove kidney stones, such as extracorporeal shock wave lithotripsy (ESWL), ureterorenoscopy (URS), retrograde intrarenal surgery (RIRS), percutaneous nephrolithotomy (PNL), and open surgery. While surgical intervention plays a pivotal role in managing urinary stones, it does have certain drawbacks, including a temporary reduction in the patient’s quality of life, prolonged hospitalization, and a high cost burden, thus favoring ESWL as a less invasive alternative. ^4^ The concept of ESWL, involving the application of shock waves to disintegrate stones, was initially introduced in Russia during the 1950s and first implemented in humans in 1980. ESWL is considered the primary choice for addressing urinary stones, particularly those smaller than 2 cm, obviating the need for surgical procedures. Success rates for stones smaller than 2 cm hover around 70-80%. However, despite its prevalence, ESWL does exhibit a reported failure rate ranging from 30% to 89% after the initial session. ^5^ Effective identification of the most suitable candidates for ESWL can substantially curtail this failure rate, thereby optimizing treatment outcomes and judiciously utilizing limited medical resources. ^3^

To discern the predictive factors affecting ESWL outcomes, numerous studies have concentrated on statistical analyses of patient characteristics employing both bivariate and multivariate approaches. Several factors have emerged as influential in determining ESWL success, including anatomical features of the urinary tract, patient age, stone location, and size. ^6^ However, it’s important to acknowledge that these estimates are derived from statistical models based on cohort data, which possess certain drawbacks. These models may overlook significant factors that fall below an arbitrarily chosen numerical threshold, typically 5%. Moreover, as these models employ dichotomized values, clinicians often encounter challenges when attempting to integrate this prognostic information into routine clinical practice.

**Figure 1.**
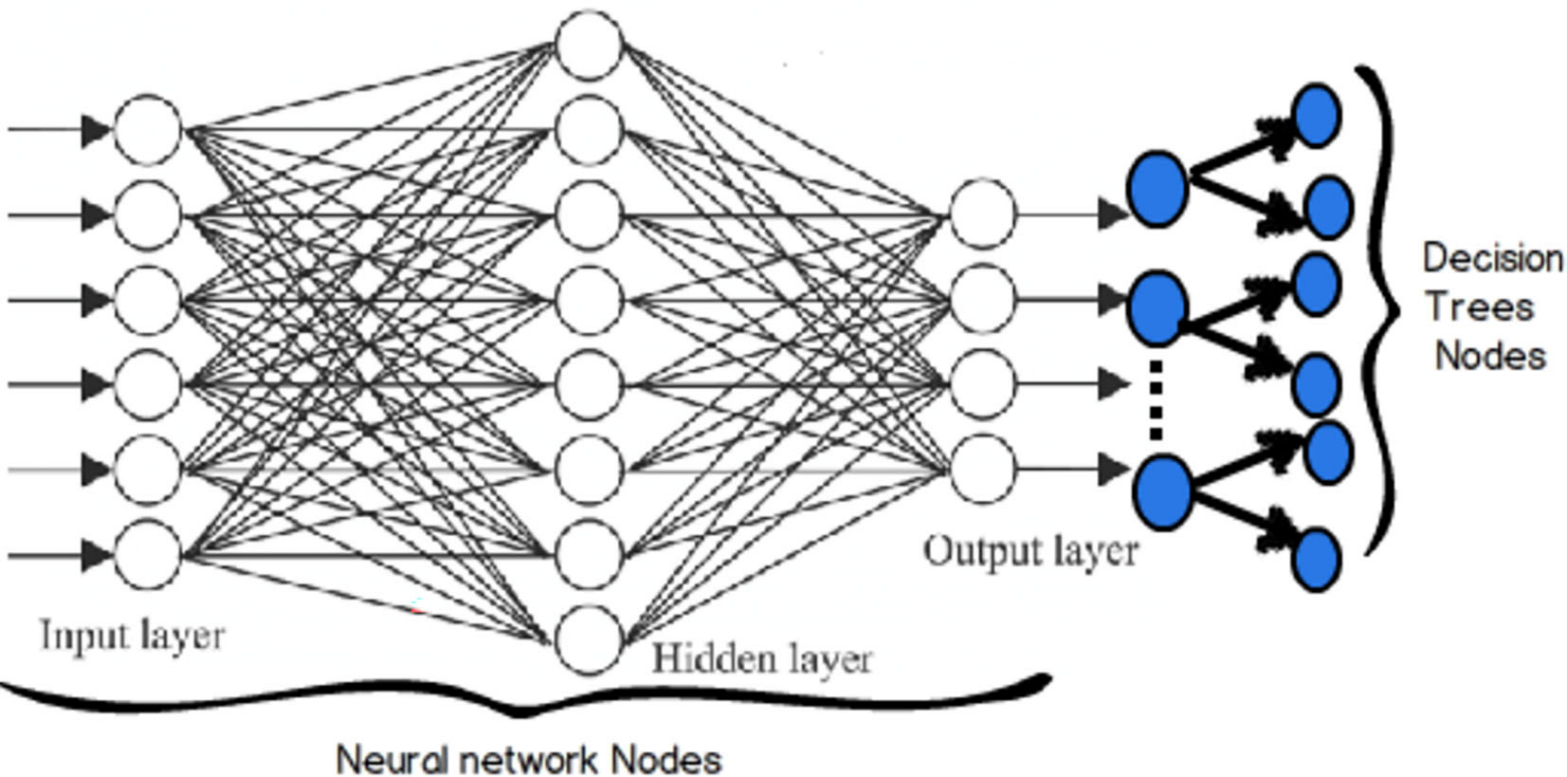
Illustration of some of the machine learnings. ^11^

In recent times, machine learning (ML) algorithms, a subset of artificial intelligence, have found increasing utility in the field of medicine. They are being widely applied to enhance the precision of disease diagnosis and prognosis. Essentially, machine learning comprises a set of techniques that enable computers to acquire knowledge from existing data sets, allowing them to make predictions. What makes machine learning particularly powerful is its capacity to rapidly analyze complex combinations of multiple variables. Among these machine learning algorithms, decision tree analysis (DTA) holds several advantages for medical applications. It is known for its simplicity in understanding and interpretation, as well as its suitability for both qualitative and quantitative data, whether textual or numeric. ^7^ Another noteworthy approach is the artificial neural network (ANN), a computational method inspired by biological brains, which approximates how biological neurons tackle problems through interconnected clusters and axons. Each neuron performs a summation function on input values, and the system is self-learning and adaptive, not relying on explicit programming. ANN excels in areas where conventional computer programs struggle to articulate solutions or recognize patterns. Once trained, the network can generate appropriate outputs for given inputs, even when confronted with patterns it has never encountered before. ANN is widely employed as an artificial intelligence technology across various domains. ^8^ Random Forests (RF) consist of an ensemble of multiple decision trees working together to establish output classifications. Numerous decision trees are trained using input data, and rather than relying exclusively on the single best-performing tree, a group of them is utilized collectively. Some of the MLs are illustrated in Figure 1. This approach is akin to a committee making decisions collectively, which often results in greater prediction accuracy compared to a single decision tree. ^9, 10^

The aim of our systematic review is to evaluate the available ML methods capable of predicting the stone-free status in patients with urolithiasis following ESWL.

## Methods

### Condition and intervention

Our objective is to assess the precision of artificial intelligence or machine learning in forecasting stone-free status in individuals with urolithiasis who have undergone ESWL treatment. Hence, this comprehensive review encompasses investigations that delve into machine learning techniques, including neural networks, to assist in computerized urography for the prediction of stone-free outcomes.

### Database searching and literature screening

A study search was conducted in six electronic databases (CENTRAL, MEDLINE, EMBASE, EBSCO, Proquest, SCOPUS) on December 12 ^th^ 2022. PICO was used to make study tracking and finding the relevant literature easier. Specific keywords were used and adapting them as needed for each database ( Table 1). References from relevant systematic reviews were also explored. To be eligible for inclusion in this systematic review, studies were limited to those available in either Indonesian or English.

**Table 1.** Search strategy.

Database	Keywords	Result	Date and time of attempt
Cochrane Central Register of Controlled Trials (CENTRAL)	Shockwave lithotripsy AND artificial intelligence	2	December 31 ^st^ 2022
MEDLINE	((urinary tract calculi OR urinary lithiasis OR urolithiasis OR kidney stone OR nephrolithiasis) AND (machine learning OR deep learning OR artificial intelligence OR AI)) AND (extracorporeal shockwave lithotripsy OR ESWL OR SWL OR shockwave lithotripsy)	96	December 31 ^st^ 2022
EMBASE	(‘shock wave lithotripsy’ OR ‘extracorporeal shock wave lithotripsy’ OR ‘eswl’:ti,ab,kw) AND (‘artificial intelligence’ OR ‘machine learning’:ti,ab,kw)	28	December 31 ^st^ 2022
EBSCO	(kidney stones or nephrolithiasis or renal calculi) AND (extracorporeal shock wave lithotripsy OR ESWL) AND (artificial intelligence or ai or a.i.)	26	December 31 ^st^ 2022
ProQuest	(“Nefrolithiasis” OR “Kidney stone” OR “urolithiasis”) AND (“Extracorporeal Shock Wave Lithotripsy” OR “ESWL”) AND (“Machine Learning” OR “Artificial intelligence”)	63	December 31 ^st^ 2022
SCOPUS	TITLE-ABS-KEY ((kidney AND stone OR urolithiasis OR nephrolithiasis) AND (extracorporeal AND shockwave AND lithotripsy OR eswl) AND (artificial AND intelligence OR are OR machine AND learning OR deep AND learning))	4	December 31 ^st^ 2022

**Study selection**

For this systematic review, we followed the guidelines provided by the Preferred Reporting Items for Systematic Reviews and Meta-Analysis (PRISMA) statements and the Standards for Reporting Diagnostic Accuracy Studies (STARD). We included randomized controlled trials (RCTs) or cohort studies that investigated the role of machine learning (ML) in predicting the stone-free status following extracorporeal shock wave lithotripsy (ESWL) for urinary system stones. These studies were required to report sensitivity, specificity, accuracy, or ROC (Receiver Operating Characteristic) data. Studies conducted on non-human subjects or those not available in English/Indonesian or without complete full-texts were excluded. Each author (NR, FF, PB, WD) independently assessed the eligibility of studies by reviewing their titles and abstracts and conducting a thorough analysis of the full texts. Any discrepancies among the authors were resolved through discussion.

**Extraction of data and outcome of interest**

Each author independently collected data using a predefined data extraction form. We gathered information on study characteristics, including patient demographics, sample sizes, and the specific machine learning techniques utilized, along with their diagnostic parameters. In cases of disagreements, consensus was reached through discussion.

The primary objective of this systematic review is to evaluate the accuracy of AI/machine learning in diagnosing stone-free status. Our main outcome measures of interest are the sensitivity, specificity, and overall accuracy of AI/machine learning when compared to the diagnostic capabilities of urography alone. We employed the Review Manager 5.4 application to gather these outcomes and score the risk of bias in the included literatures.

**Methodological assessment**

This systematic review encompasses diagnostic investigations employing both experimental and observational study designs. To gauge the potential bias in all the studies, we employed the Quality Assessment of Diagnostic Accuracy Studies (QUADAS-2) tool.

## Results

### Literature search

**Figure 2.**
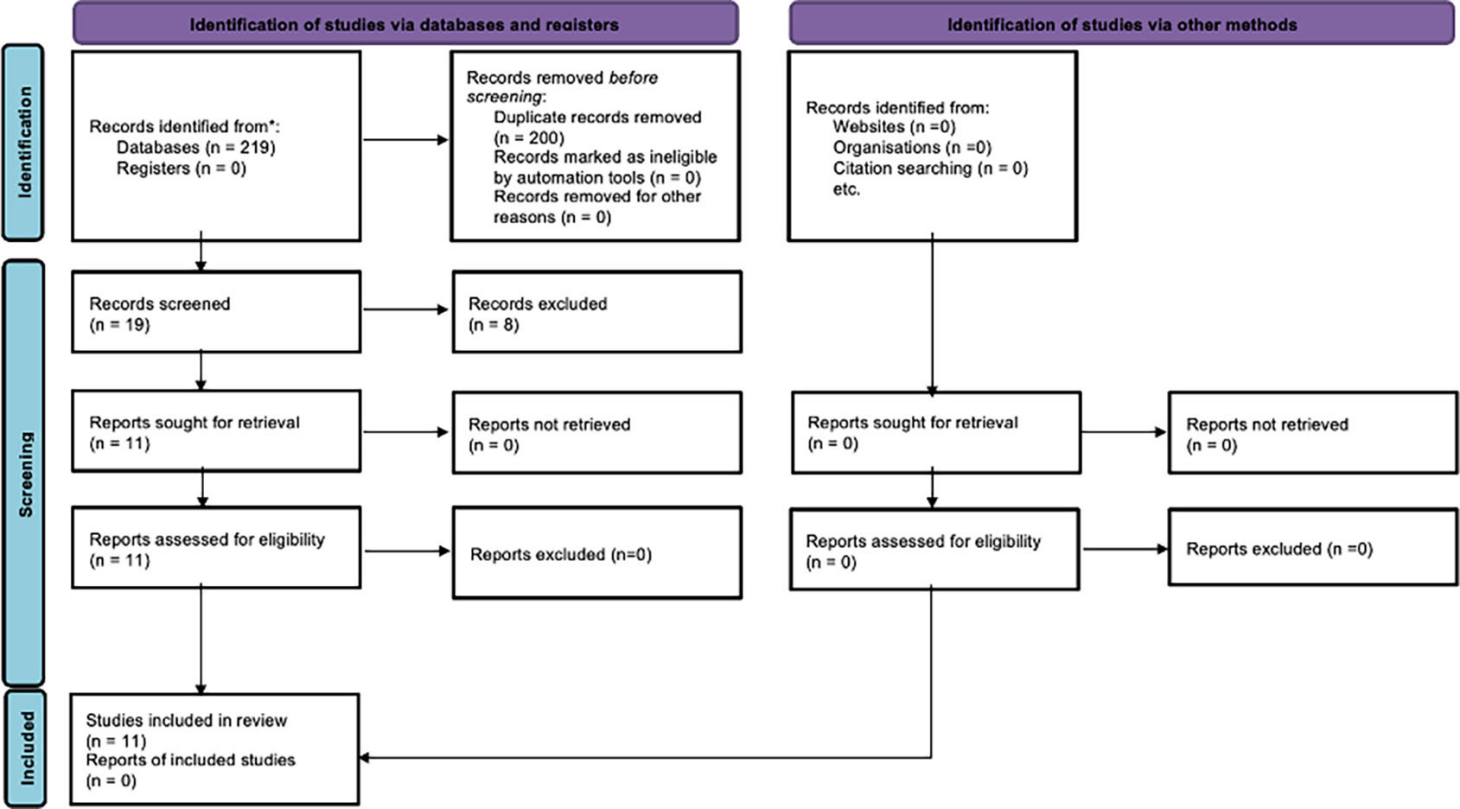
PRISMA flow chart of including articles.

The initial search across six electronic databases yielded a total of 219 articles, of which 200 were duplicates. After screening the titles and abstracts of the remaining articles, we identified 19 studies that aligned with the criteria set by our systematic review’s PICO framework. However, upon conducting a thorough analysis of the full-text articles, only eleven met our PICO criteria, while the remaining eight studies did not meet the inclusion criteria for this review ( Figure 2).

### Study characteristics

Eleven studies were included in this systematic review. Based on the population of each study, this systematic review involved 41,484 patients. These studies are spread across four continents, Europe, Asia, America, and Africa, most of which are conducted in Asia. The characteristics of the studies are summarized in Table 2.

**Table 2.** Summary of evidence.

Author (Year)	Factors included in model	Stone Location	Stone Size (mm)	Machine Learning Model	Total sample (Training/Test) (n)	Outcomes	Sensitivity	Specificity	Accuracy	ROC AUC
Michaels EK ^12^ et al. (1998)	• Previous stone events • Metabolic abnormality (Hypercalciuria, hyperuricosuria) • Infection • **Stone size** • Directed medical therapy • Caliectasis/dilated collecting system • Residual fragments after ESWL	Kidney, Ureter	N/A	ANN	98 (65/33)	Stone-free status three months after treatment through abdominal radiographs	91%	92%	91%	0.964
Poulakis V ^13^ et al. (2003)	• Age • BMI • No SWL sessions • SWL intensity • No of stones • **Stone size** • Stone surface area • Infundibular length • Infundibular diameter • Caliceal pelvic height • Lower infundibulopelvic angle • Infundibulouretero-pelvic angle • **Urinary transport type**	Kidney (Lower pole)	14.79 ± 6.53	ANN	701 (600/101)	Stone-free status after six months	91%	90%	92%	N/A
Hamid A ^14^ et al. (2003)	• Clinical (Age, Body Habitus) • 24-hour urinary volume • **Size of stones** • Hydronephrosis • ESWL (total number of sittings, total number of shocks, mean power, mean frequency)	Kidney	N/A	ANN	82 (60/22)	Stone-free status during 1.5 years of the study period through Intravenous Urogram (IVU)	N/A	N/A	75%	N/A
Gomha MA ^15^ et al. (2004)	• Age • Sex • Kidney Side • Stone Location • Stent • Renal Anatomy • Stone nature • Stone number • **Stone length** • Stone width	Ureter	N/A	ANN	984 (688/296)	Stone-free status three months after treatment through urinary tract plain x-ray and excretory urography	77.9%	75%	77.7%	N/A
Moorthy K ^16^ et al. (2016)	• The mean of grey level matrix	Kidney	10-20	ANN	120 (80/40)	Stone-free status one month after treatment through non-contrast enhance computed tomography (NCCT)	80.7%	98.4%	90%	N/A
Seckiner I ^3^ et al. (2017)	• Gender • No of stone • Location • Infundibulopelvic angle (IPA) • Primary/secondary nature of stone • Status of hydronephrosis • Age • Skin-to-stone distance • **Stone density** • Creatinine level	Kidney	N/A	ANN	203 (171/32)	Stone free status	N/A	N/A	88.7%	N/A
Mannil M ^10^ et al. (2018)	• **Three-dimensional texture analysis (3D-TA)**	Kidney	5-20	J48 Decision tree kNN ANN RF SMO	51 (34/17)	Stone-free status 83 days after treatment by non-contrast abdominal CT scans	71% 53% 65% 71% 0.35	74% 68% 72% 74% 0.63	N/A	0.72 0.61 0.6 0.79 0.49
Choo MS ^7^ et al. (2018)	• Age • Gender • Location • Length • Width • **Stone Volume** • **Stone Length** • Skin-to-stone distance • **Stone Density** • Creatinine • Urine specific gravity • pH • Urinalysis microscopic RBC	Ureter	5.9 ± 2.3	DTA	791 (791/0)	Stone-free status two weeks after treatment through CT scans or plain x-ray of kidneys, ureters, and bladder	0.96	0.86	0.92	0.951
Yang SW ^9^ et al. (2020)	• **Mean stone density** • **Stone volume** • Skin-to-stone distance • Stone length • Psoas muscle cross-sectional area	Kidney Ureter	9.2 ± 3.5 6.6 ± 1.5	RF XGBoost LightGBM	358 (286/72)	Stone-free status (4,5 and 6 weeks after treatment, respectively) by non-contrast CT scans	0.74 0.75 0.78	0.92 0.93 0.92	0.86 0.87 0.88	0.85 0.84 0.85
Xu ZH ^8^ et al. (2021)	• **Stone length** • Duration of diseased • Age • Stone width • pH	Ureter	N/A	ANN	1083 (813/270)	Stone-free status three months after treatment by KUB radiography	N/A	N/A	93.2%	0.935
Moghisi R ^17^ et al. (2022)	• **Stone location** • Age • Kidney side • Electrode used • Stone treatment number • Number of shocks • Area of stone • Gender • BMI • Number of stones • Family history	Kidney Ureter	N/A	AdaBoost	37013 (37013/0)	Stone-free status three months after treatment	87.5%	65.3%	77.59%	0.843

Abbreviations: ANN: Artificial Neural Network; DTA: Decision Tree Analysis; kNN: k-nearest neighbor; RF: Random Forest; SMO: Sequential Minimal Optimization; XGBoost: Extreme Gradient Boosting Trees; LightGBM: Light Gradient Boosting Method; BMI: Body Mass Index.

### Risk of bias

**Figure 3.**
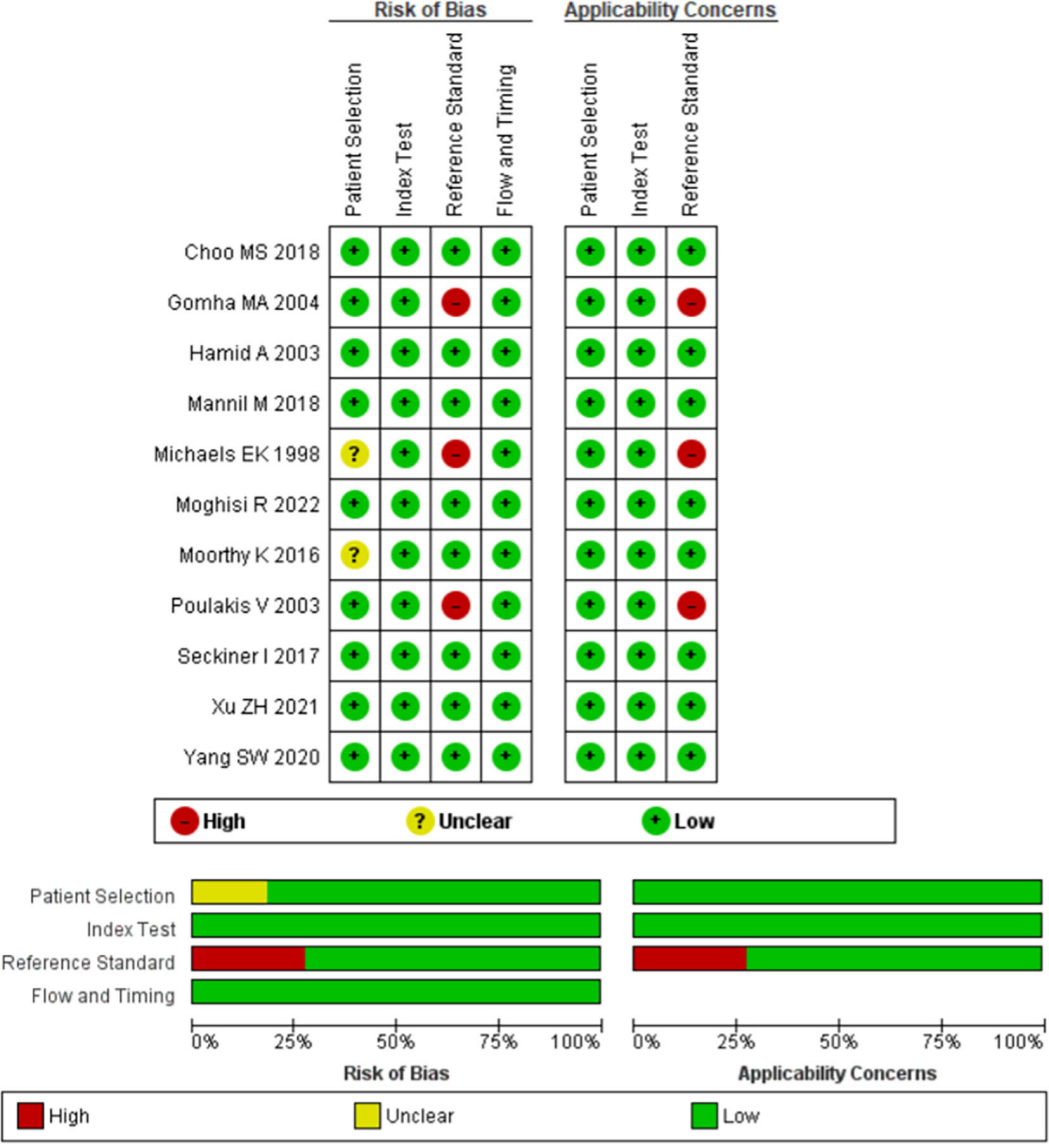
Risk of bias assessment using Cochrane Risk of Bias Assessment.

This systematic review analyzes the accuracy of machine learning in predicting a condition; therefore, diagnostic articles are included. We use the Non-Proprietary Quality Assessment of Diagnostic Accuracy Studies APPRAISE-AI tool to assess the quality of the diagnostic studies. ^18^ Out of 11 included studies, 2 are moderate quality, 6 are high quality and 3 are very high. The risk of bias assessment is shown in Supplementary Table 1. Generally, the included studies are of moderate to good quality, only three of which have moderate quality. The risk of bias assessment is shown in Figure 3.

Only two studies reported the use of additional procedures. Yang et al. performed an additional SWL session every one week if stones remained. ^9^ Postoperative stents, catheters, and contrast medium were used by Gomha et al. ^15^ In three articles, the absence of stones on plain abdominal radiographs determined stone-free status. ^12, 13, 15^ Two studies did not state their sampling method. ^12, 16^

### Artificial Intelligence/Machine Learning Accuracy in Predicting Stone-free Status

A total of 9 MLs are observed in 11 studies, which are ANN, J48 decision tree, k-nearest neighbor (kNN), RF, Sequential Minimal Optimization (SMO), DTA, Extreme Gradient Boosting Trees (XGBoost), Light Gradient Boosting Method (LightGBM), and AdaBoost. ^7– 9^ Eight out of 11 studies explored the diagnostic parameters of ANN. Most studies report that ANN has high specificity and high overall accuracy in predicting stone-free status. Four experiments out of 8 studies demonstrated ANN accuracy of at least 90%, with its accuracy ranging from 75-93%. It is followed by DTA with the accuracy of 92% and LightGBM with the accuracy of 88%.

## Discussion

This review employed broad search criteria and inclusive inclusion criteria to ensure the inclusion of all relevant studies. Based on the Area Under the Curve (AUC) values, the ANN model presented by Michaels et al. ^12^ achieved the highest AUC. However, it’s noteworthy that this study utilized plain abdominal radiography as the reference standard instead of computerized urography. Interestingly, when plain abdominal radiography is used as the reference standard, the specificity tends to be higher, particularly when the stone is located outside the kidneys. Decision support systems like ANN serve as computer-generated algorithms aiding healthcare professionals in clinical decision-making. These algorithms, in various forms, aim to replicate the learning process of the human brain. They assist healthcare practitioners in making decisions based on specific clinical data from patients. By employing functions within ANN, computers are trained to predict specific parameters efficiently using training sets. Following training, the computer’s performance is evaluated using the data, assessing the extent of its learning in terms of validity and test data. If the computer demonstrates sufficient learning, it can make predictions for users. ^3^

ANN has the potential to greatly enhance the quality of healthcare services, facilitating early disease detection, reducing medical errors, and supporting healthcare authorities in cost-effective patient care. The decision-making process involves selecting one of several alternative outcomes generated by cognitive functions. With an increasing number of alternatives, the complexity of the decision-making process and the potential for errors also rise. At the conclusion of the decision-making process, there is an action or idea. Different formats can be employed to depict how individuals arrive at decisions. It is imperative to scrutinize the interplay between psychological elements, cognitive processes, and the surrounding context when assessing the decision-making process. Individuals are expected to generate specific recommendations through logical filtering and ultimately arrive at the correct decision. Given the value of time in decision-making, providing decision-makers with data as quickly as possible is essential for effective and rapid decision-making. Consequently, many administrative and specialized organizations currently rely on ANN for efficient and swift decision-making. ^3^

Machine learning models exhibit variable diagnostic accuracies, with sensitivity ranging from 35% to 96%, specificity from 63% to 98.4%, and AUC of ROC ranging from 0.49 to 0.96. In this study, it is demonstrated that the predictive accuracy of Random Forest (RF) and Decision Tree Analysis (DTA) in determining stone-free status surpasses that of ANN. It’s worth noting that the high AUC value of ANN was partly attributed to the use of plain abdominal radiography as a reference standard in some studies. Decision tree models present numerous benefits compared to current decision support tools. In contrast to decision tools relying on statistical approaches, artificial intelligence decision models adjust their operations based on the data as they are employed, allowing for the smooth incorporation of new data. Although this adaptability may pose overfitting challenges, setting a minimum number of cases can address this issue. Decision tree models can handle data with both quantitative and qualitative variables, making them versatile. Additionally, results are presented in a straightforward and interpretable manner, often in the form of a tree or a set of rules. Nonetheless, there are certain drawbacks to consider, like attribute importance metrics potentially exhibiting a bias toward variables possessing higher levels of data, including categorical variables with differing value counts. ^7^ RF, XGBoost, and LightGBM are three models based on DTA, explaining their comparable accuracy. ^9^

Information derived from the analysis of CT images consistently includes crucial predictive factors in the models, primarily mean stone density, stone volume, length, width, and three-dimensional texture analysis. These findings align with current guidelines. ^19^ Specifically, stone size, volume, length, or width are considered essential predictive factors in eight studies, while stone density is emphasized in three studies, and three-dimensional texture analysis is featured in one study. Incorporating these factors into the model enhances the predictive capability of machine learning for stone-free status.

Several steps are involved in developing AI models. Some of the programs utilized in these studies include Alyuda NeuroIntelligence 2.2 for creating ANN and Quinlan C.50 for producing DTA. ^3, 7^ The algorithm parameters were set to default values to identify factors with the highest predictive accuracy. In the case of DTA, the “minimal cases” parameter defaults to 2 and is used to restrict splits at nodes, leading to the creation of smaller trees as the value increases. By reducing splits at nodes based on this parameter, accuracy gradually decreases, preventing overfitting.7 During the creation of ANN, data were analyzed concerning training, validation, and testing. Both numerical and categorical data were used, and the percentage of data allocated to training, validation, or testing was determined. Subsequently, all data were converted into a numeric format for processing. The ANN structure was then formulated, with the number of neurons determined experimentally, as there are no specific rules in the literature for determining this. The logistic activation function was applied to all inputs and outputs in the ANN model, transforming values into the 0-1 range. ^3^ The RF model, developed by Mannil ^10^ et al., was created using open-source data mining software (WEKA, version 3.8.0; University of Waikato, Hamilton, New Zealand). A built-in feature selection filter was employed to assess individual features’ value and predictive contribution to SWL (Shock Wave Lithotripsy) success, considering both unique predictive value and correlation between features. ^10^

This systematic review has several limitations. Many included studies had small sample sizes in both training and testing sets, potentially misrepresenting the AI's true capabilities. The data were predominantly retrospective, introducing potential bias. Methodological issues, including reproducibility, the use of test datasets, diagnostic accuracy reporting, and lack of non-ML statistical comparisons, were also noted. The reproducibility of most studies is limited, with only one study providing model access. This limits the ability to evaluate models with different datasets. There is also significant variability in ESWL outcomes, highlighting the need for consensus to reduce heterogeneity. Furthermore, none of the studies compared their machine learning methods with clinical experts' evaluations, which could provide insights into the effectiveness of AI in clinical settings.

Therefore, future studies should use bigger, prospective, and multicenter data that are externally validated with increased methodological transparency to develop better AI-models. Hybrid models by combining multiple ML approaches should also be explored to improve these existing models. Incorporating standardized reporting of ESWL outcomes predictors will made more progress to increase its clinical applicability.

## Conclusion

In conclusion, ML can be used for predicting stone-free status in urinary stone diseases with satisfying accuracy however the accuracy of the prediction rely on many factors such as the ML model, variables taken into account and the data used for training set. The main advantage of ML in the prediction of stone free-status was it allows various factors to be taken into consideration even with the non-linear variables. Random forest method and DTA are superior MLs compared to ANN. Stone size, density, and 3D-texture analysis should be included in the models to ensure accuracy in predicting stone-free status after ESWL. However, access to the models should be made public, and further studies comparing them to the current statistical methods should be conducted.

## Data Availability

Figshare: Artificial Intelligence or Machine learning for Prediction of stone-free status in Patients with urolithiasis.rm5, http://doi.org/10.6084/m9.figshare.27082798. ^20^ The project contains the following underlying data:
• Artificial Intelligence or Machine learning for Prediction of stone-free status in Patients with urolithiasis.rm5 (57.68 kB) Data are available under the terms of the Creative Commons Attribution 4.0 International license (CC-BY 4.0). Figshare: Artificial Intelligence in the Prediction of Stone-Free Status in Urinary Stone Disease Treated with Extracorporeal Shockwave Lithotripsy: A Systematic Review, DOI: http://doi.org/10.6084/m9.figshare.27173805, ^21^ http://doi.org/10.6084/m9.figshare.27314595 ^22^ The project contains the following Reporting guidelines data:
• PRISMA Checklist • Prisma Flow Chart Data are available under the terms of the Creative Commons Attribution 4.0 International license (CC-BY 4.0).
