# Supplementary material for: Artificial Intelligence in the Prediction of Stone-Free Status in Urinary Stone Disease Treated with Extracorporeal Shockwave Lithotripsy: A Systematic Review
Source: F1000Res. 2025 Mar 26;14:16. Originally published 2025 Jan 3. [Version 2] doi: 10.12688/f1000research.152346.2 (PMC12498514; doi:10.12688/f1000research.152346.2)
Supplement: Peer review history

# Reviewer report: Reviewer response for version 2

**DOI:** 10.5256/f1000research.179593.r410676

**Published:** 2025-09-18

**Authors:** Shriyansh Chahar

**Affiliations:** 1 Wims Hospital, Agra, India, Agra,, India

This systematic review examines the application of artificial intelligence (AI) and machine learning (ML) models in predicting stone-free outcomes following extracorporeal shockwave lithotripsy (ESWL). The authors conducted a comprehensive search across six databases, included 11 studies (41,484 patients), and assessed methodological quality using PRISMA, STARD, QUADAS-2, and APPRAISE-AI frameworks. The review highlights that Random Forest (RF) and Decision Tree Analysis (DTA) models outperform artificial neural networks (ANN) in prediction accuracy, and identifies stone size, density, and 3D texture analysis as key predictive variables.

 The study is timely, relevant, and well-structured. The authors have addressed most concerns raised in previous reviews, including updating the search, applying APPRAISE-AI, clarifying stone-free status definitions, and expanding the limitations section.

 A few points merit attention. First, the heterogeneity of included studies—differences in stone-free definitions, patient cohorts, and ML algorithms—should be emphasized more clearly, with consideration for subgroup analysis or at least a stronger acknowledgment of its impact on comparability.

 Second, while comparisons with statistical models are made, there is no benchmarking against clinician judgment; highlighting this gap would strengthen the clinical relevance.

 Third, the discussion on hybrid/ensemble approaches could be expanded, as combining ML models may improve predictive power.

 The limitations section could also be refined by distinguishing between weaknesses of the included studies (retrospective design, small datasets, lack of validation) and limitations of the review itself (language restriction, inability to perform meta-analysis).

 Lastly, simplifying Table 2 or adding a summary figure would improve readability.

Are the rationale for, and objectives of, the Systematic Review clearly stated?

Yes

Is the statistical analysis and its interpretation appropriate?

Yes

If this is a Living Systematic Review, is the ‘living’ method appropriate and is the search schedule clearly defined and justified? (‘Living Systematic Review’ or a variation of this term should be included in the title.)

Yes

Are sufficient details of the methods and analysis provided to allow replication by others?

Yes

Are the conclusions drawn adequately supported by the results presented in the review?

Yes

Reviewer Expertise:

endourology, urolithiasis

I confirm that I have read this submission and believe that I have an appropriate level of expertise to confirm that it is of an acceptable scientific standard, however I have significant reservations, as outlined above.

# Reviewer report: Reviewer response for version 2

**DOI:** 10.5256/f1000research.179593.r373263

**Published:** 2025-03-31

**Authors:** Ranjit Barua

**Affiliations:** 1 Indian Institute of Engineering Science and Technology, Howrah, West bengal, India

I have re-reviewed the manuscript. The authors have modified the paper according to the suggestions.

Are the rationale for, and objectives of, the Systematic Review clearly stated?

Yes

Is the statistical analysis and its interpretation appropriate?

Yes

If this is a Living Systematic Review, is the ‘living’ method appropriate and is the search schedule clearly defined and justified? (‘Living Systematic Review’ or a variation of this term should be included in the title.)

Yes

Are sufficient details of the methods and analysis provided to allow replication by others?

Yes

Are the conclusions drawn adequately supported by the results presented in the review?

Yes

Reviewer Expertise:

Modern Robotics, MIS, Bioprinting

I confirm that I have read this submission and believe that I have an appropriate level of expertise to confirm that it is of an acceptable scientific standard.

# Reviewer report: Reviewer response for version 1

**DOI:** 10.5256/f1000research.167095.r361858

**Published:** 2025-01-30

**Authors:** Ranjit Barua

**Affiliations:** 1 Indian Institute of Engineering Science and Technology, Howrah, West bengal, India

Every scientific study has limitations, and acknowledging them strengthens its credibility. The current study should explicitly discuss potential drawbacks, such as small sample sizes, retrospective data analysis, and lack of external validation. Addressing these limitations would help guide future research in refining ML models for ESWL outcome prediction.

 The potential for hybrid models should also be explored. Combining multiple ML approaches, such as ensemble learning techniques that integrate RF and ANN, could improve predictive accuracy. Additionally, discussing the need for prospective, multi-center trials to validate ML models in real-world clinical settings would provide a clear roadmap for future research.

 The study presents valuable insights into the application of ML in predicting ESWL outcomes, but several areas require further refinement to enhance its scientific soundness. Methodological transparency, proper statistical validation, assessment of bias, and clinical applicability must be addressed to ensure the reliability and utility of ML models in urological practice. By implementing these recommendations, the study can contribute more effectively to the ongoing integration of ML into clinical decision-making for urolithiasis treatment.

Are the rationale for, and objectives of, the Systematic Review clearly stated?

Yes

Is the statistical analysis and its interpretation appropriate?

Yes

If this is a Living Systematic Review, is the ‘living’ method appropriate and is the search schedule clearly defined and justified? (‘Living Systematic Review’ or a variation of this term should be included in the title.)

Yes

Are sufficient details of the methods and analysis provided to allow replication by others?

Yes

Are the conclusions drawn adequately supported by the results presented in the review?

Yes

Reviewer Expertise:

Modern Robotics, MIS, Bioprinting

I confirm that I have read this submission and believe that I have an appropriate level of expertise to confirm that it is of an acceptable scientific standard, however I have significant reservations, as outlined above.

# Response

**Published:** 2025-03-21

**Authors:** Ficky Huang

**Affiliations:** Dr. Cipto Mangunkusumo Hospital, Indonesia

1. Acknowledging Limitations: We have acknowledged the limitations of small sample sizes, retrospective data, and lack of external validation. We’ve emphasized that future research should focus on larger, prospective, multi-center studies to better validate machine learning models.

 2. Exploring Hybrid Models: In response to the suggestion, we’ve included the idea of exploring hybrid models combining Random Forest and Artificial Neural Networks (ANN). This could enhance predictive accuracy, and we plan to consider this approach in future studies.

 3. Methodological Transparency and Clinical Applicability: We’ve agreed on the importance of transparency in methodology and comparing machine learning models with clinical experts’ evaluations. We highlighted the need for standardized outcome reporting and reducing variability in ESWL outcomes to improve clinical applicability.

# Reviewer report: Reviewer response for version 1

**DOI:** 10.5256/f1000research.167095.r358874

**Published:** 2025-01-24

**Authors:** Jethro CC Kwong

**Affiliations:** 1 University of Toronto, Toronto, Canada

1. The search was conducted on Dec 12, 2022. I would recommend updating the search to at least Dec 31, 2024 as there may be newer models published in the past 2 years

 2. This review can be strengthened by examining the quality of the included ML studies using an AI-specific evaluation tool, such as APPRAISE-AI (Kwong JCC, et al. [2023-Ref -1] https://doi.org/10.1001/jamanetworkopen.2023.35377). It would be worthwhile to use this tool to identify specific methodological and reporting gaps in this specific area of study.

 3. Did any of the studies compare the performance of the ML model against a non-ML approach (clinical judgement or other clinical tool)? If yes, what was the margin of difference? If not, this may be worthwhile to highlight as these studies did not demonstrate whether the ML approach outperforms traditional statistical approaches or clinical judgement

 4. The authors should provide more detail regarding the stone-free status. From Table 2, it seems that there was heterogeneity in the timepoint at which stone-free status was determined, which can impact comparability of studies. Furthermore, additional details should be provided regarding determination of stone-free status (e.g. imaging modality, does stone-free mean complete absence of stone or was there a size cutoff of residual stone that was allowed).

 5. The limitations of the included studies should be expanded. I think use of the APPRAISE-AI tool will help uncover additional limitations that are worth mentioning. This review can be strengthened if the authors can provide a list of recommendations on how future ML studies in this space can be improved, based on the limitations identified in the current review.

Are the rationale for, and objectives of, the Systematic Review clearly stated?

Yes

Is the statistical analysis and its interpretation appropriate?

Partly

If this is a Living Systematic Review, is the ‘living’ method appropriate and is the search schedule clearly defined and justified? (‘Living Systematic Review’ or a variation of this term should be included in the title.)

Not applicable

Are sufficient details of the methods and analysis provided to allow replication by others?

Partly

Are the conclusions drawn adequately supported by the results presented in the review?

Partly

Reviewer Expertise:

AI in urology

I confirm that I have read this submission and believe that I have an appropriate level of expertise to confirm that it is of an acceptable scientific standard, however I have significant reservations, as outlined above.

## References

[1] : APPRAISE-AI Tool for Quantitative Evaluation of AI Studies for Clinical Decision Support. *JAMA Netw Open* .2023;6(9) : 10.1001/jamanetworkopen.2023.35377 e2335377 10.1001/jamanetworkopen.2023.35377 37747733 PMC10520738

# Response

**Published:** 2025-03-21

**Authors:** Ficky Huang

**Affiliations:** Dr. Cipto Mangunkusumo Hospital, Indonesia

**1. The search was conducted on Dec 12, 2022. I would recommend updating the search to at least Dec 31, 2024 as there may be newer models published in the past 2 years**

 Response: Updating the Search: We’ve decided to update the search to include studies published up to December 31, 2024, to ensure the review stays current with the latest advancements in machine learning for predicting stone-free status.

**2. This review can be strengthened by examining the quality of the included ML studies using an AI-specific evaluation tool, such as APPRAISE-AI (Kwong JCC, et al. [2023-Ref -1] https://doi.org/10.1001/jamanetworkopen.2023.35377). It would be worthwhile to use this tool to identify specific methodological and reporting gaps in this specific area of study.**

 Response: AI-Specific Evaluation Tool (APPRAISE-AI): We’ve recognized the value of using the APPRAISE-AI tool to assess the quality of machine learning studies. We have used APPRAISE-AI tool and made some changes.

**3. Did any of the studies compare the performance of the ML model against a non-ML approach (clinical judgement or other clinical tool)? If yes, what was the margin of difference? If not, this may be worthwhile to highlight as these studies did not demonstrate whether the ML approach outperforms traditional statistical approaches or clinical judgement**

 Response: Comparison with Non-ML Approaches: In response to the feedback, we will include a focus on comparing machine learning models with traditional methods, like clinical judgment, to evaluate their effectiveness in clinical practice.

**4. The authors should provide more detail regarding the stone-free status. From Table 2, it seems that there was heterogeneity in the timepoint at which stone-free status was determined, which can impact comparability of studies. Furthermore, additional details should be provided regarding determination of stone-free status (e.g. imaging modality, does stone-free mean complete absence of stone or was there a size cutoff of residual stone that was allowed).**

 Response: Clarification on Stone-Free Status: We’ve addressed the variability in defining stone-free status across studies and committed to providing more clarity on how this status was determined, including the imaging modalities used.

**5. The limitations of the included studies should be expanded. I think use of the APPRAISE-AI tool will help uncover additional limitations that are worth mentioning. This review can be strengthened if the authors can provide a list of recommendations on how future ML studies in this space can be improved, based on the limitations identified in the current review.**

 Response: Expanding the Limitations: We expanded the limitations section further and apply the APPRAISE-AI tool to uncover additional issues, with recommendations for improving future machine learning studies.
